# Editorial: Supramolecular Aspects in Catalysis

**DOI:** 10.3389/fchem.2019.00174

**Published:** 2019-03-26

**Authors:** Pablo Ballester, Alessandro Scarso

**Affiliations:** ^1^Institute of Chemical Research of Catalonia (ICIQ), Barcelona Institute of Science and Technology, Tarragona, Spain; ^2^Institució Catalana de Recerca i Estudis Avançats (ICREA), Barcelona, Spain; ^3^Dipartimento di Scienze Molecolari e Nanosistemi, Università Ca' Foscari di Venezia, Venezia Mestre, Italy

**Keywords:** supramolecular chemistry, supramolecular catalysis, molecular recognition, non-covalent interactions, self-assembly

When a catalyst and its substrate get in contact, they become involved in a series of weak attractive interactions that are preparatory to the real catalytic event that is the stabilization of an elusive target: the transition state. These weak and reversible interactions are responsible, among other functions, of the substrate's preorganization, orientation, and activation. From the initial substrate-catalyst complex, and through all the catalytic cycle, supramolecular interactions are at work in shaping the entire chemical transformation. Recently, the term supramolecular catalysis (Wei and Wang, 2013; Lin et al., 2014) was coined to refer to a resurrected cross discipline aiming at bridging the gap between classic homogeneous catalysis and the enzymatic catalysis. A goal in this area consists on the development of synthetic host structures capable of displaying modulation of chemical reactivity or even catalytic activity and turn-over. Many host structures (Deraedt and Astruc, 2016) can display supramolecular catalytic properties (Raynal et al., 2014a,b) exploiting their recognition properties (Leclercq et al., 2019), especially when these features enable the stabilization of intermediate species or transition states.

The main objective of this research topic is to showcase, to the largest possible audience, the importance of considering weak intermolecular forces when dealing with substrate selectivity, product selectivity, improving the turnover number of a homogeneous catalyst. Basically, supramolecular catalysis consists on the rational use of weak and reversible interactions to improve and control homogeneous catalysis at all levels. Moreover, molecular confinement within rigid or conformationally flexible nano-environments is an alternative method to impart further control on catalytic activity and selectivity.

Overall, this research topic consists of four contributions. In particular, three are reviews that covers the main aspects of supramolecular catalysis. Two major approaches to supramolecular catalysis are treated and discussed by the authors of the reviews. One of them covers the confinement effects in catalysis. Molecular, covalent, solid, as well as non-covalent supramolecular host structures endowed with catalytic features are presented. The other one, deals with catalysis in water with micelles or macrocyclic units. It also serves as a link between homogeneous catalysis and enzymatic catalysis because the use of water in chemical transformations implies the presence of the hydrophobic effect, which often is responsible for unique features.

Supramolecular catalysis is always connected with substrate recognition in terms of shape, size and presence of specific functional groups to engage in weak intermolecular forces. This confinement effect is present in heterogeneous catalysis, in metal organic frameworks capable of catalytic performance and in covalent molecular containers for catalysis like cyclodextrins, calix[n]arenes, cucurbit[n]urils, and self-assembled supramolecular containers. The importance and the positive effects of confinement in catalysis is the subject of the review paper by Mouarrawis et al. titled “Confinement Effects in Catalysis Using Well-Defined Materials and Cages.” The take home message of the work is to look at catalysis in a general sense, crossing the borders between homogeneous and heterogeneous systems, just considering, at a molecular level, what the substrate would experience during its interaction with the catalyst.

A simple yet efficient way to mimic the catalytic effects of enzymes in water as a green and unusual reaction medium consists on using micelles self-assembled from simple surfactants or surfactants endowed with catalytic units. In particular, the apolar core of the micelles is responsible for the confinement effects and the superficial polar units are responsible for the catalytic effects. The confinement effects of micelles are spurred by the hydrophobic effect that ensures high local concentrations of several organic substrates in the nanometric environment of micelles. This is a key ingredient of micellar catalysis applied to multicomponent reactions described in the review paper by Paprocki et al. titled “multicomponent reactions accelerated by aqueous micelles.” This review covers examples over the last 20 years for common multicomponent reactions, as well as many other unusual transformations for the synthesis of complex heterocycles.

The hydrophobic effect can be exploited as a supramolecular driving force in water. This constitutes the topic of the third review contribution in the research topic, which is entitled “Supramolecular Organocatalysis in Water Mediated by Macrocyclic Compounds” by De Rosa et al. The review paper focus on recent catalytic applications in water of macrocycles like cyclodextrins and calixarenes. Specifically, cyclodextrins can promote multicomponent reaction and thanks to their chirality, products can be obtained in some cases with a high level of stereoselectivity as well as regioselectivity. While cyclodextrins are intrinsically water soluble, calixarenes can promote some chemical transformations under “on water” conditions which means that the reactants and the catalyst are not completely soluble in water and the hydrophobic effect pushes the organocatalyst to interact with the substrates thus promoting the reactions. The article underlines also important features of cyclodextrins in catalysis like the regiochemistry of transformations as well as the enantioselectivity for cyclodextrins bearing catalytically active chiral groups.

Finally, one intriguing research article underlying the potentialities of a large self-assembled hexameric capsule has been provided by Köster et al. The authors reported in their contribution the use of the well-known resorcin[4]arene hexameric capsule for the activation of alkyl fluorides. Generally, the transformation of the alkyl fluorides into valuable products requires the use of harsh acidic conditions and the presence of Lewis of Brønsted acids. Alternatively, in organic media, the use of the self-assembled hexameric capsule in catalytic amounts, as low as 10 mol%, and at a temperature of 40°C promotes the conversion of primary and secondary benzylic and tertiary alkyl (sp^3^)C-F bonds through encapsulation of the substrates. Different classes of products are obtained through the formation of new C-C and C-O bonds. A combination of weak interactions such as, hydrogen bonding together with the stabilization of cationic intermediates are key to the catalytic function of the container.

In conclusion, we hope that this research topic will help to shape future directions in supramolecular catalysis. The development of new, efficient and selective supramolecular catalysts will spur our understanding of separate pieces of information. In turn, this development will be useful for improving our understanding of enzymatic catalysis, and eventually leading to the realization of synthetic enzymes.

## Author Contributions

All authors listed have made a substantial, direct and intellectual contribution to the work, and approved it for publication.

### Conflict of Interest Statement

The authors declare that the research was conducted in the absence of any commercial or financial relationships that could be construed as a potential conflict of interest.
